# High-efficiency and low-hazard artillery recoil reduction technology based on barrel gas reflection

**DOI:** 10.1038/s41598-024-58313-2

**Published:** 2024-03-29

**Authors:** Fu He, Jinsong Dai, Shengye Lin, Maosen Wang, Xiaopeng Su

**Affiliations:** 1 School of Mechanical Engineering, Nanjing University of Science and Technology, Nanjing, 210094 China; 2 Chongqing Changan Wangjiang Industry Group Co., Ltd., Chongqing, 401120 China

**Keywords:** Mechanical engineering, Engineering, Fluid dynamics

## Abstract

Reducing recoil as well as reducing muzzle hazards are important issues in artillery design. This paper presents a barrel gas reflection method for the artillery aiming for efficient recoil reduction while reducing muzzle hazards. The launching process is modeled by coupling the interior ballistic equations and the flow equations of the barrel gas reflection device. The fourth-order Runge–Kutta method was used to solve the model, and the LHS method as well as the Kriging model was used to establish a mapping relationship between the parameters and the effect. To validate the proposed model, shooting experiments are carried out on a 30 mm caliber artillery. The maximum error between the experiment and simulation results was 5.32%. The experiment has demonstrated that the barrel gas reflection method increases the recoil reduction efficiency of artillery by 44.54% and reduces the muzzle hazard by 52.18%. Finally, the barrel gas reflection method can produce effects with the muzzle device at the same time, and it has little effect on the velocity of the projectile muzzle velocity, and it provides a new way of thinking for the development of future artillery recoil reduction technology.

## Introduction

For continuous-fire artillery, when firing, the enormous energy generated by the burning of gunpowder not only accelerates the projectile but also creates a huge recoil force on the artillery structure, and a recoil motion is induced. Studies^1–4^ have shown that such recoil and recoil motion if left unchecked, can bring about strong vibrations, damage or shorten the fatigue life of artillery components, limit the frequency of artillery fire, and reduce the mobility of the artillery system. Along with the projectile flying out of the muzzle, the high-temperature, high-pressure, and high-speed gunpowder gas will also be discharged from the muzzle, and the resulting flame and shock wave will be a great hazard to the equipment and personnel around the artillery.^5^ In addition, the muzzle flame and shock waves easily expose the location of the firing position to the enemy.

By far, a large number of articles have been published on the study of reducing artillery recoil, and various schemes have been used in their research, such as increasing artillery recoil displacement^6^, controlling artillery firing charge^7^, optimizing the structure and parameters of the recoil buffer^8^, opening the chamber in advance^9^, adopting the principle of Davis artillery^10^, and adopting the principle of recoilless artillery^11–14^, among other schemes. All of these schemes can provide a good recoil reduction effect but also will be accompanied by the creation of new problems, such as making the size and weight of the artillery larger, making the kinetic energy of the projectile lower, reducing the firing frequency of the artillery, and reducing the recoil inefficiently, and so on, and so these schemes can only be applied to a specific artillery structure. Among the many schemes for reducing artillery recoil, the method of reducing artillery recoil by utilizing the thrust generated by the propellant gases can be applied to most cases, and the efficiency of this method in reducing recoil is high, so this method has been most widely used. The muzzle brake is the most representative application of this method^15–17^. However, the complex muzzle brake structure will make the already complex muzzle flow field even more complex, and the disturbance of the muzzle flow field has a great influence on the projectile attitude^5,15^, which will affect the firing accuracy of the artillery. At the same time, the muzzle flow field created by the muzzle brake that spreads to the side and rear can also exacerbate the muzzle hazard when the artillery is fired. The most common way to mitigate the muzzle hazard is to add a muzzle flame trap^18,19^, however, the structure of a muzzle flame trap is complicated and the recoil reduction effect produced by a muzzle flame trap is much lower than that of a muzzle brake. In summary, it is necessary to study how to efficiently reduce recoil while reducing muzzle hazards.

This paper proposes a method of utilizing gas reflection from the barrel of artillery to achieve high efficiency in reducing recoil while reducing muzzle hazards. Combined with the established physical model, the dynamic model of firing is developed by coupling the interior ballistic equations and the flow equations for the barrel gas reflection device, combined with the artillery dynamics model, and the model is solved by using the fourth-order Runge–Kutta method. To verify the proposed model, an experiment for testing the recoil reduction efficiency, muzzle velocity, and muzzle pressure is carried out. Finally, the effects of the location of the orifice, the orifice diameter, and the length of the barrel gas reflection device on the artillery are analyzed systematically.

## Barrel gas reflection method and theoretical model

**Figure 1:**
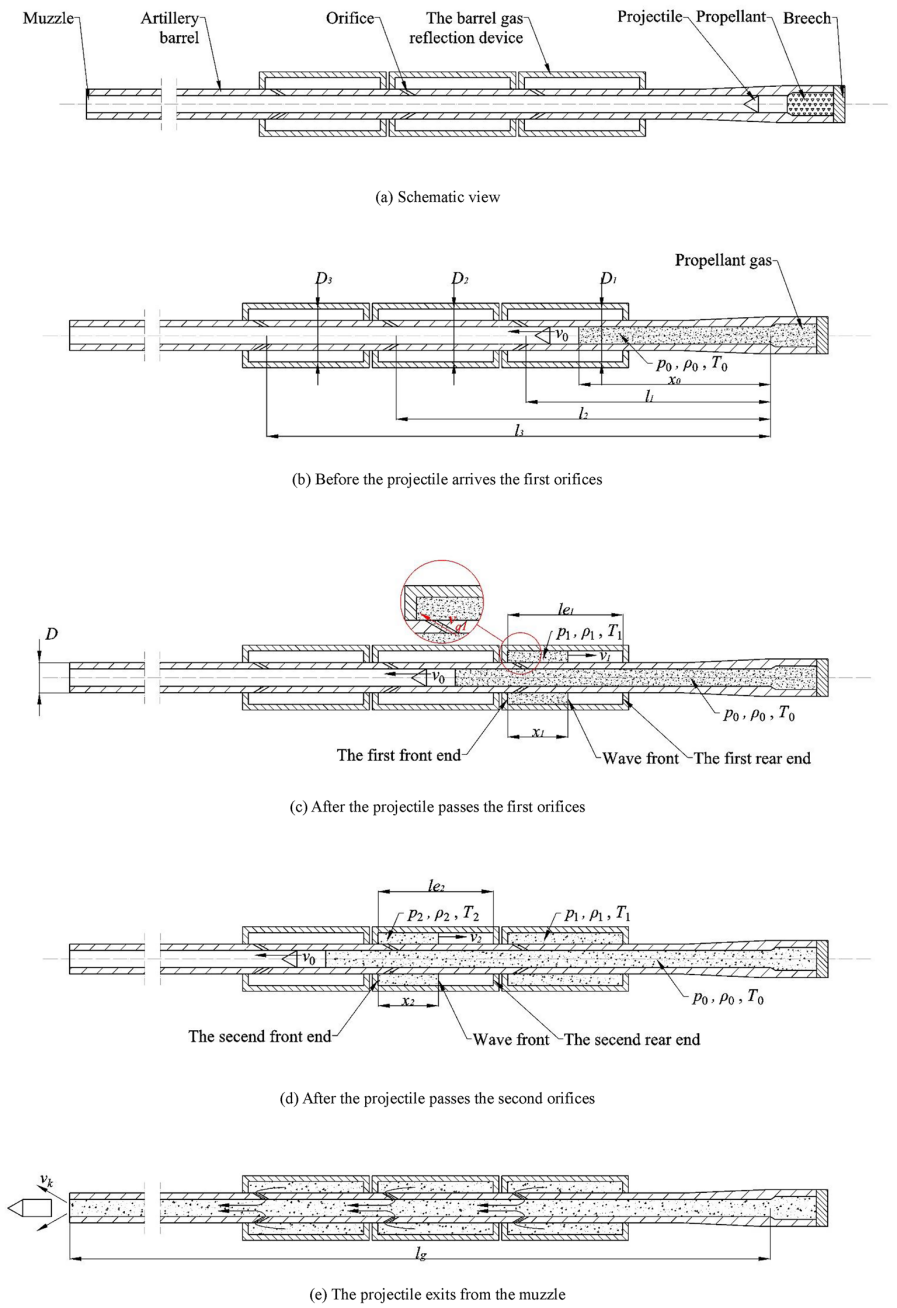
Schematic view of the artillery barrel equipped with the barrel gas reflection device and the launching process. (**a**) Schematic view. (**b**) Before the projectile arrives the first orifices. (**c**) After the projectile passes the first orifices. (**d**) After the projectile passes the second orifices. (**e**) The projectile exits from the muzzle.

Figure 1 is a schematic view of the principle of utilizing exported barrel gases to efficiently reduce recoil while reducing muzzle hazards. Figure 1a shows the schematic structure of the principle. The barrel gas reflection device is equipped in the middle of the barrel, and the device forms several cavities (3 in the figure) with the outer wall of the barrel. Each cavity of the device is connected to the artillery bore through several orifices, which are straight in shape. After the projectile is fired, the high-pressure gas pushes the projectile forward in the barrel, as shown in Fig. 1b, and this process is no different from that of conventional artillery. When the projectile passes through the first orifices, as shown in Fig. 1c, the high-pressure gas will flow through the orifices into the first cavity of the barrel gas reflection device, creating a high-pressure zone at the front of the first cavity and reflecting backward, which creates a forward thrust against the recoil motion of the artillery. When the reflected wave front in the first cavity reaches the rear end of the cavity, the first cavity of the barrel gas reflection device is no longer capable of reducing recoil. The projectile continues to move forward during this process, and when the first cavity of the barrel gas reflection device is disabled, the projectile passes through the second orifices and the second cavity starts to work, as shown in Fig. 1d. The subsequent cavities of the device work on a similar principle as the first two cavities and serve to extend the effective time of the barrel gas reflection device and increase the effective reflective area of the high-pressure gas. When the projectile leaves the muzzle, the high-pressure gas follows the outflow, the pressure of the artillery bore drops drastically, and the gas in the barrel gas reflection device flows back into the artillery bore and is then discharged from the muzzle, as shown in Fig. 1e.

### Assumption

To formulate the dynamic model for the artillery launching process with the barrel gas reflection device, the following assumptions are made:

1. The flow within the artillery bore and the barrel gas reflection device is considered one-dimensional, quasi-steady, and thermally isolated.
2. The passage of the projectile through the orifices is assumed to occur within an extremely short time, thus disregarding the opening process of the orifices.
3. The wave propagation within the cavity of the barrel gas reflection device is assumed to travel to the rear end at the local speed of sound.
4. The propellant gas is assumed to exit through the muzzle at the local speed of sound.

### Dynamic model

The dynamic model for the artillery equipped with the barrel gas reflection device can be obtained by coupling the interior ballistic model^20,21^ and the flow equations for the barrel gas reflection device, which are as follows:

(1) Form function of the propellant

1 $$ \psi { = }\left\{ {\begin{array}{*{20}l} {\chi z\left( {1 + \lambda z + \mu z^{2} } \right)} \hfill & {z < 1} \hfill \\ {\chi_{s} \frac{z}{{z_{k} }}\left( {1 + \mu_{s} \frac{z}{{z_{k} }}} \right)} \hfill & {1 \le z < z_{k} } \hfill \\ 1 \hfill & {z \ge z_{k} } \hfill \\ \end{array} } \right. $$

where, $\psi$ and $z$ denote the relative burned mass and thickness of the propellant respectively; $\chi$, $\lambda$, $\mu$, $\chi_{s}$ and $\mu_{s}$ are form characteristic parameters of the propellant. The propellant grain used in this study is seven-hole gunpowder.

(2) Burning equation of the propellant

2 $$ \frac{dz}{{dt}} = \left\{ {\begin{array}{*{20}l} {\frac{{u_{0} }}{{e_{0} }}p_{0}^{n} } \hfill & {z < z_{k} } \hfill \\ 0 \hfill & {z \ge z_{k} } \hfill \\ \end{array} } \right. $$

where, $u_{0}$, $n$ and $e_{0}$ denote the burning rate coefficient, burning rate-pressure exponent, and half the thickness of the combustible layer of the propellant grain respectively; $p_{0}$ denotes the average bore pressure; $t$ denotes the time.

(3) Travel equation of the projectile

3 $$ \frac{{dx_{0} }}{dt} = v_{0} $$

where, $x_{0}$ and $v_{0}$ denote the travel and velocity of the projectile respectively.

(4) Motion equation of the projectile

4 $$ \left\{ {\begin{array}{*{20}c} {\varphi m\frac{{dv_{0} }}{dt} = S_{0} p_{0} } & {x_{0} < l_{g} } \\ {\varphi m\frac{{dv_{0} }}{dt}{ = }0} & {x_{0} \ge l_{g} } \\ \end{array} } \right. $$

where, $S_{0}$ denotes the cross-section area of the artillery bore; $\varphi$ denotes the coefficient of the secondary works in the artillery bore; $l_{g}$ denotes the barrel length; $m$ denotes the projectile mass.

(5) Energy equations for the artillery bore

5 $$ \left\{ {\begin{array}{*{20}l} {p_{0} S_{0} \left( {l_{\psi } + x_{0} } \right){ = }f\omega \psi - \frac{1}{2}\theta \varphi mv_{0}^{2} } \hfill & {x_{0} < l_{1} } \hfill \\ {p_{0} S_{0} \left( {l_{\psi } + x_{0} } \right){ = }f\omega \psi - \frac{1}{2}\theta \varphi mv_{0}^{2} - \theta \sum\limits_{i = 1}^{{n_{1} }} {\int {e_{i} q_{i} dt} } } \hfill & {l_{1} \le x_{0} < l_{g} } \hfill \\ {p_{0} S_{0} \left( {l_{\psi } + l_{g} } \right){ = }f\omega \psi - \frac{1}{2}\theta \varphi mv_{0}^{2} - \theta \left( {\sum\limits_{i = 1}^{{n_{1} }} {\int {e_{i} q_{i} dt} } + \int {e_{k} q_{k} dt} } \right)} \hfill & {x_{0} \ge l_{g} } \hfill \\ \end{array} } \right. $$

6 $$ l_{\psi } = l_{0} \left[ {1 - \frac{\Delta }{{\rho_{p} }} - \Delta \left( {\alpha - \frac{1}{{\rho_{p} }}} \right)\psi } \right]\,\,{\text{with}}\,\,l_{0} = \frac{{V_{0} }}{{S_{0} }}\,\,{\text{and}}\,\,\Delta = \frac{\omega }{{V_{0} }} $$

7 $$ \theta = k - 1 $$

where, $l_{\psi }$ denotes the equivalent length of the free chamber volume; $l_{0}$ denotes the equivalent length of the chamber; $V_{0}$ denotes the chamber volume; $\Delta$ denotes the loading density; $\omega$ denotes the charge mass; $\alpha$ denotes the covolume of the propellant gas; $\rho_{p}$ denotes the propellant density; $f$ denotes the propellant force; $k$ denotes the specific heat ratio; $n_{1}$ denotes the number of cavities; $l_{i} \left( {i = 1,2,...,n_{1} } \right)$ denotes the distance between the orifices of the ith cavity and the chamber throat; $q_{i} \left( {i = 1,2,...,n_{1} } \right)$ and $q_{k}$ denote the flow rate per second through the orifices of the ith cavity and the muzzle respectively; $e_{i} \left( {i = 1,2,...,n_{1} } \right)$ and $e_{k}$ denote the energy per unit mass of the propellant gas through the orifices of the ith cavity and the muzzle respectively, which are expressed as follows:

8 $$ e_{i} = \left\{ {\begin{array}{*{20}c} {C_{p} T_{0} } & {p_{0} \ge p_{i} } \\ {C_{p} T_{i} } & {p_{0} < p_{i} } \\ \end{array} } \right. $$

9 $$ e_{k} = C_{p} T_{0} $$

where, $C_{p}$ denotes the specific heat at constant pressure; $p_{i} \left( {i = 1,2,...,n_{1} } \right)$ denotes the average pressure in the ith cavity of the barrel gas reflection device; $T_{0}$ and $T_{i} \left( {i = 1,2,...,n_{1} } \right)$ denote the temperature of the propellant gas in the artillery bore and the ith cavity of the barrel gas reflection device respectively.

(6) Equations of gas state

10 $$ p_{0} \left( {\frac{1}{{\rho_{0} }} - \alpha } \right) = RT_{0} $$

11 $$ p_{i} = \rho_{i} RT_{i} $$

where, $\rho_{0}$ and $\rho_{i} \left( {i = 1,2,...,n_{1} } \right)$ denote the density of the propellant gas in the artillery bore and the ith cavity of the barrel gas reflection device respectively; $R$ denotes the gas constant.

(7) Energy equation for the ith cavity of the barrel gas reflection device

12 $$ e_{i} q_{i} = \frac{{dU_{i} }}{dt} $$

where, $U_{i} \left( {i = 1,2,...,n_{1} } \right)$ denotes the potential energy of the propellant gas in the ith cavity of the barrel gas reflection device, which can be calculated by using the following equations:

13 $$ U_{i} = \rho_{i} V_{i} C_{V} T_{i} $$

14 $$ C_{V} = \frac{1}{k - 1}R $$

where, $V_{i} \left( {i = 1,2,...,n_{1} } \right)$ denotes the volume of the propellant gas in the ith cavity of the barrel gas reflection device; $C_{V}$ denotes the specific heat at constant volume.

By substituting Eqs. (11), (13) and (14) into Eq. (12), one can obtain:

15 $$ \frac{{dp_{i} }}{dt} = \frac{1}{{V_{i} }}\left( {\theta e_{i} q_{i} - p_{i} \frac{{dV_{i} }}{dt}} \right) $$

(8) Continuity equations

For the artillery bore:

16 $$ \frac{{dm_{0} }}{dt}{ = }\omega \frac{d\psi }{{dt}} - \sum\limits_{i = 1}^{{n_{1} }} {q_{i} } - q_{k} $$

where, $m_{0}$ denotes the mass of the propellant gas in the artillery bore, it can also be expressed as:

17 $$ m_{0} { = }\rho_{0} \left( {l_{\psi } + x_{0} } \right)S_{0} $$

By substituting Eq. (17) into Eq. (16), one can obtain:

18 $$ \frac{{d\rho_{0} }}{dt}{ = }\frac{{\left( {\omega - \rho_{0} S_{0} l_{0} \frac{\Delta }{{\rho_{p} }}} \right)\frac{d\psi }{{dt}} - \sum\limits_{i = 1}^{{n_{1} }} {q_{i} } - q_{k} - \rho_{0} S_{0} v_{0} }}{{\left( {l_{\psi } + x_{0} } \right)S_{0} }} $$

For the ith cavity of the barrel gas reflection device:

19 $$ \frac{{dm_{i} }}{dt}{ = }q_{i} $$

where, $m_{i} \left( {i = 1,2,...,n_{1} } \right)$ denotes the mass of the propellant gas in the ith cavity of the barrel gas reflection device, it can also be expressed as:

20 $$ m_{i} { = }\rho_{i} V_{i} $$

By substituting Eq. (20) into Eq. (19), one can obtain:

21 $$ \frac{{d\rho_{i} }}{dt}{ = }\frac{{q_{i} - \rho_{i} \frac{{dV_{i} }}{dt}}}{{V_{i} }} $$

(9) Wave velocity equation

22 $$ \frac{{dx_{i} }}{dt}{ = }\left\{ {\begin{array}{*{20}l} {\sqrt {\frac{{kp_{i} }}{{\rho_{i} }}} } \hfill & {x_{i} < le_{i} } \hfill \\ 0 \hfill & {x_{i} = le_{i} } \hfill \\ \end{array} } \right. $$

where, $x_{i} \left( {i = 1,2,...,n_{1} } \right)$ denotes the displacement of the wave front in the ith cavity of the barrel gas reflection device, $le_{i} \left( {i = 1,2,...,n_{1} } \right)$ denotes the length of the ith cavity of the barrel gas reflection device.

(10) Flow equations.

For the orifices:

23 $$ q_{i} = \left\{ {\begin{array}{*{20}l} {\mu_{o} S_{i} \left( {\frac{2}{k + 1}} \right)^{{\frac{k + 1}{{2\left( {k - 1} \right)}}}} \sqrt {k\rho_{0} p_{0} } } \hfill & {p_{i} \le \left( {\frac{{2}}{k + 1}} \right)^{{\frac{k}{k - 1}}} p_{0} } \hfill \\ {\mu_{o} S_{i} \sqrt {\frac{2k}{{k - 1}}\rho_{0} p_{0} \left[ {(\frac{{p_{i} }}{{p_{0} }})^{\frac{2}{k}} - (\frac{{p_{i} }}{{p_{0} }})^{{\frac{k + 1}{k}}} } \right]} } \hfill & {\left( {\frac{{2}}{k + 1}} \right)^{{\frac{k}{k - 1}}} p_{0} < p_{i} < p_{0} } \hfill \\ { - \mu_{o} S_{i} \sqrt {\frac{2k}{{k - 1}}\rho_{i} p_{i} \left[ {(\frac{{p_{0} }}{{p_{i} }})^{\frac{2}{k}} - (\frac{{p_{0} }}{{p_{i} }})^{{\frac{k + 1}{k}}} } \right]} } \hfill & {\left( {\frac{{2}}{k + 1}} \right)^{{\frac{k}{k - 1}}} p_{i} < p_{0} < p_{i} } \hfill \\ { - \mu_{o} S_{i} \left( {\frac{2}{k + 1}} \right)^{{\frac{k + 1}{{2\left( {k - 1} \right)}}}} \sqrt {k\rho_{i} p_{i} } } \hfill & {p_{0} \le \left( {\frac{{2}}{k + 1}} \right)^{{\frac{k}{k - 1}}} p_{i} } \hfill \\ \end{array} } \right. $$

where, $S_{i} \left( {i = 1,2,...,n_{1} } \right)$ denotes the equivalent area of the ith orifices; $\mu_{o}$ denotes the discharge coefficient of the orifices.

For the muzzle:

24 $$ q_{k} = \mu_{k} S_{0} \sqrt {k\rho_{0} p_{0} } $$

where, $\mu_{k}$ denotes the discharge coefficient of the muzzle.

### Recoil reduction efficiency

The fourth-order Runge–Kutta method is adopted to solve the dynamic model, and the interior ballistic characteristics are obtained. After that, the recoil characteristics of artillery can be expressed as:

25 $$ \left\{ \begin{gathered} \frac{dx}{{dt}} = u \hfill \\ \frac{du}{{dt}} = \frac{{F_{pt} - F_{R} }}{M} \hfill \\ \end{gathered} \right. $$

where, $t$ denotes time;$x$ denotes the recoil displacement of artillery; $u$ denotes the recoil velocity of artillery;$M$ denotes the recoil mass of artillery;$F_{pt}$ denotes the combining forces of the barrel;$F_{R}$ denotes recoil of the artillery. $F_{pt}$ and $F_{R}$ are expressed as follows:

26 $$ F_{pt} = p_{0} S_{0} - \sum\limits_{i = 1}^{{n_{1} }} {p_{i} S_{i} } $$

27 $$ F_{R} = F_{0} + Kx + Ru^{2} $$

where, $F_{0}$, $K$ and $R$ denote the initial force, the stiffness, and the damping of the recoil buffer respectively, they are related to the specific construction and material of the recoil buffer.

The recoil reduction efficiency of the barrel gas reflection device can be obtained by using the following equation:

28 $$ \eta = \frac{{F_{0} \left( {X_{0} - X} \right) + \frac{1}{2}K\left( {X_{0}^{2} - X^{2} } \right)}}{{F_{0} X_{0} + \frac{1}{2}KX_{0}^{2} }} $$

where, $X_{0}$ and $X$ denote the recoil displacement of the conventional artillery and the artillery equipped with the barrel gas reflection device.

## Results and discussion

### Effect of the barrel gas reflection device

Based on the proposed model, a 30 mm caliber artillery equipped with the barrel gas reflection device with three cavities is analyzed. Parameters of the 30 mm caliber artillery are listed in Table 1. In the table, $d_{1}$, $d_{2}$, $d_{3}$ and refer to the diameters of the three groups of orifices, respectively. Besides, a conventional 30 mm caliber artillery is also simulated for comparison, to reveal the barrel gas reflection device on the interior ballistic characteristics of the artillery.

**Table 1:** Parameters of the 30 mm caliber artillery equipped with the barrel gas reflection device.

Item	Value	Item	Value	Item	Value	Item	Value
$l_{g}$ (m)	2.3	$l_{1}$ (m)	1.000	$l_{2}$ (m)	1.225	$l_{3}$ (m)	1.450
$d_{1}$ (m)	0.01	$d_{2}$ (m)	0.01	$d_{3}$ (m)	0.01	$S_{0}$ (m^2^)	7.38 × 10^–4^
$D_{1}$ (m)	0.092	$D_{2}$ (m)	0.092	$D_{3}$ (m)	0.092	$D$ (m)	0.052
$le_{1}$ (m)	0.2	$le_{2}$ (m)	0.2	$le_{3}$ (m)	0.2	$V_{0}$ (m^3^)	1.25 × 10^–4^
$\alpha$ (m^3^/kg)	0.001	$\rho_{p}$ (kg/m^3^)	1600	$\varphi$	1.09	$f$ (J/kg)	1.05 × 10^6^
$k$	1.25	$\omega$ (kg)	0.1	$m$ (kg)	0.3	$M$ (kg)	80
$F_{0}$ (N)	4000	$K$ (N/m)	68,000	$R$ (Ns^2^/m^2^)	200		

**Figure 2:**
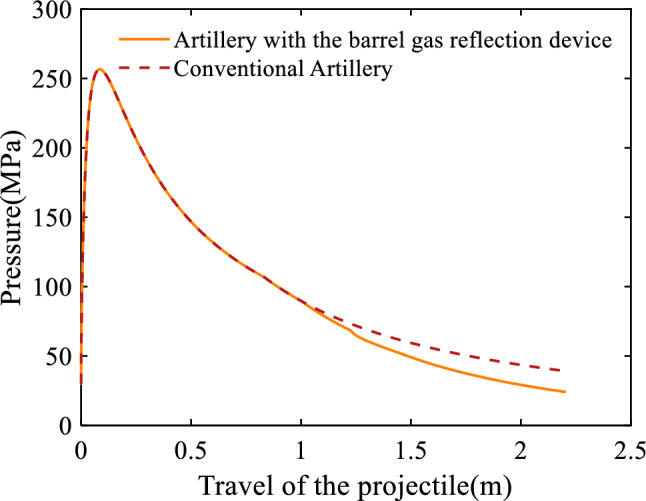
Variation of the average bore pressure with respect to the travel of the projectile.

Figure 2 shows the average bore pressure of the 30 mm caliber artillery with respect to the travel of the projectile. It can be seen that, when the travel of the projectile is less than 1.0 m, which is the distance between the breech and the first orifices, the average bore pressure of the artillery equipped with the barrel gas reflection device is the same as that of the conventional artillery; while when the travel of the projectile exceeds 1.0 m, the average bore pressure of the artillery equipped with the barrel gas reflection device decreases further than that of the conventional artillery. This is caused by the fact that the orifices are enabled when the travel of the projectile is larger than 1.0 m, the propellant gas enters the first cavity of the barrel gas reflection device through the first orifices, which leads to the further decline of the average bore pressure. Similarly, because of the presence of the second cavity and the third cavity, there is the same decline at 1.225 m and 1.450 m. It can also be seen from Fig. 2 that, the maximum bore pressure, which is 256.58 MPa, appears before the projectile arrives at the orifices; when the projectile leaves the muzzle, the average bore pressure of the artillery equipped with the barrel gas reflection device and the conventional artillery is 24.17 MPa and 39.24 MPa respectively. The results indicate that the effect of the barrel gas reflection device on the maximum bore pressure is unobvious, but the muzzle pressure was significantly reduced by 38.40%. Muzzle hazards are mainly caused by the high-temperature, high-pressure, and high-velocity gunpowder gases emitted from the muzzle after the projectile has exited the muzzle. The severity of these hazards is directly related to the muzzle pressure at the moment the projectile exits the muzzle: higher muzzle pressures correspond to greater hazards and vice versa. From the simulation results, it can be seen that the application of the barrel gas reflection device can effectively reduce the muzzle hazards. At the same time, the muzzle velocity would not decrease dramatically.

**Figure 3:**
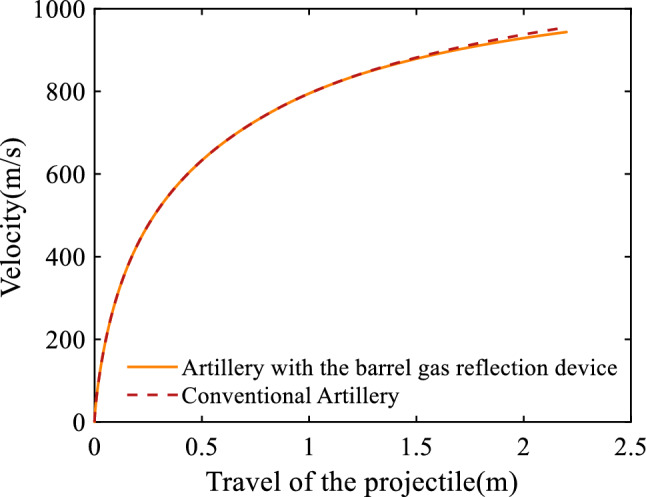
Variation of the velocity of the projectile with respect to the travel of the projectile.

Figure 3 gives the velocity of the projectile with respect to the travel of the projectile. It can be seen that when the travel of the projectile is less than 1.0 m, the velocities of the projectiles in the two artilleries are equal to each other; but when the travel of the projectile exceeds 1.0 m, the curve of the projectile in the artillery equipped with the barrel gas reflection device growth rate gets smaller. In conjunction with Fig. 2, it can be seen that the decrease in projectile velocity is due to the decrease in bore pressure as a result of the gas flow into the first cavity of the barrel gas reflection device. The same reduction occurred at 1.225 m and 1.450 m. The velocity of the projectile in the artillery equipped with the barrel gas reflection device is slightly smaller than that in the conventional artillery; the muzzle velocities of the two artilleries are 943.62 m/s and 955.06 m/s respectively. For the artillery equipped with the barrel gas reflection device, the muzzle velocity declines by just 1.20%.

**Figure 4:**
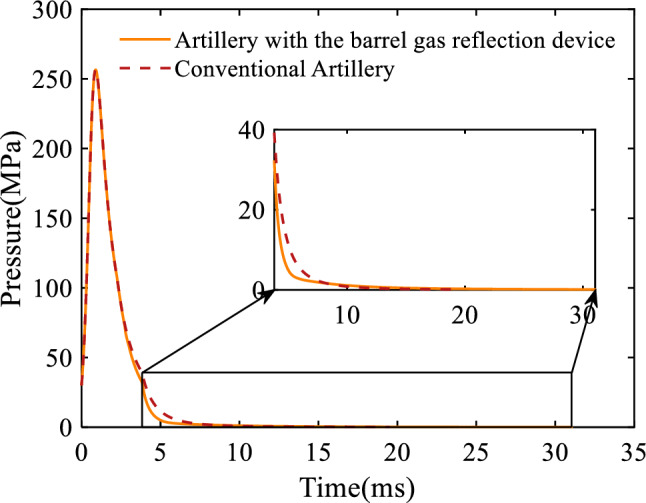
Variation of the average bore pressure with respect to time.

Furthermore, to provide insight into the working mechanism of the barrel gas reflection device, the after-effect period of artillery is included. Figure 4 shows the average bore pressure of the 30 mm caliber artillery with respect to time. It can be seen that the difference between the average bore pressure of the artillery equipped with the barrel gas reflection device and that of the conventional artillery increases first and then decreases with time. This is caused by the fact that the propellant gas flows into the cavities of the barrel gas reflection device when the average bore pressure is larger than the average pressure of the cavities; while the propellant gas flows back to the artillery bore when the average bore pressure is smaller than the average pressure of the cavity. This will result in an 11.53 ms increase in the artillery's after-effect period. For continuous-fire artillery with a firing frequency of less than 1000 shots/min, with a single-fire period of at least 60 ms, the artillery's after-effect period with the addition of the device is still less than the single-shot period of artillery, so the device does not affect the movement of the subsequent projectiles and the firing frequency of the artillery.

**Figure 5:**
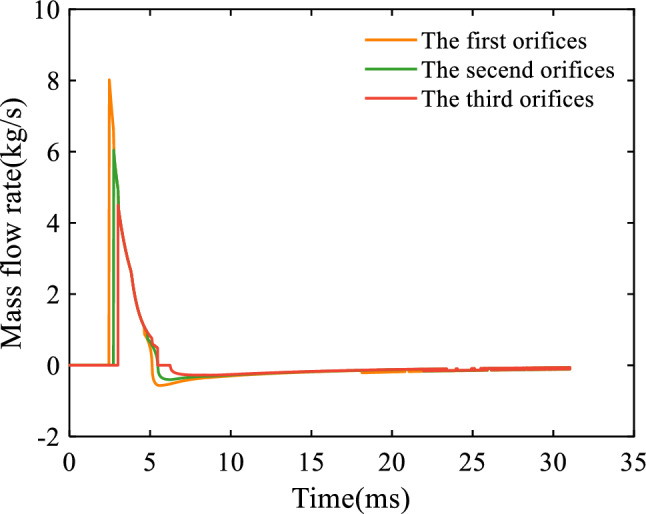
Variation of the mass flow rate through the orifices with respect to time.

**Figure 6:**
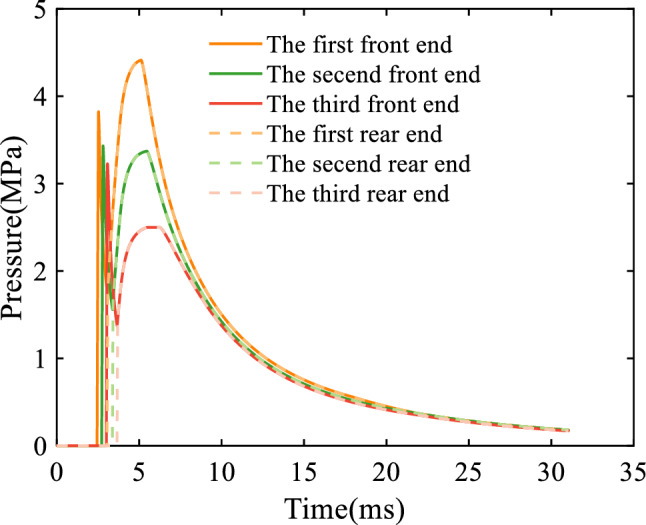
Variation of the pressure acting on the front and rear ends of the barrel gas reflection device.

Figure 5 illustrates the variation of the mass flow rate through the orifices. The plus or minus sign of the flow rate represents the direction of gas flow, with a positive value representing flow from the artillery bore to the cavity and a negative value representing flow from the cavity to the artillery bore. It can be seen that the first orifices are enabled at 2.474 ms, and the propellant gas begins to enter the cavity of the barrel gas reflection device. The mass flow rate sharply decreases with time due to the decreasing bore pressure and the increasing cavity pressure, which is likewise confirmed by Figs. 4 and 6. The mass flow rate turns to be negative at 5.124 ms, indicating that the propellant gas flows back to the artillery bore. The mass flow rate of the second and third orifices showed the same trend, except that they start later and have a smaller mass flow rate. It can be seen from Eq. (23) that when the bore pressure is much higher than the cavity pressure, the mass flow rate of the orifices almost only depends on the bore pressure. This explains the coincidence of curves up to 4.646 ms.

Figure 6 shows the change of gas pressure acting on the front end and rear end of the barrel gas reflection device, from which it can be seen that the gas pressure on the front end of the first cavity of the barrel gas reflection device suddenly increases at 2.474 ms, which indicates that the gas begins to flow into the first cavity. At this time, because the propellant gas wave front did not reach the rear end of the cavity, the cavity will provide a forward force for the artillery, which can reduce the recoil of the artillery. As the propellant gas expands backward in the device, the pressure in the cavity begins to decrease, and when the gas reaches the rear end of the cavity, the pressure at the rear end increases and equalizes with the pressure at the front end. The volume of propellant gas in the cavity no longer changes, and the pressure at the front end and rear end continues to increase (or decrease) with the inflow (or outflow) of propellant gas. The gas pressure change rule of the second cavity and the third cavity is the same as that of the first cavity, only the pressure value and change time are different. This is because the bore pressure decreases over time, which is shown in Fig. 4.

**Figure 7:**
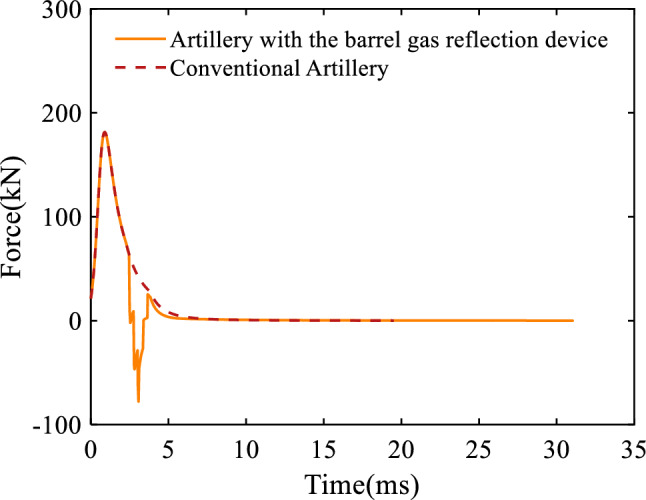
Variation of the combined force of the propellant gas with respect to time.

Figure 7 represents the relationship between the combined force of the propellant gas acting on the artillery as a function of time, including the force of the propellant gas acting in the artillery bore and the force acting on the barrel gas reflection device, with the positive and negative of the force indicating the direction, positive being the direction of recoil. It can be seen that the maximum combined force of the artillery equipped with the barrel gas reflection device is the same as that of the conventional artillery. However, for the artillery equipped with the barrel gas reflection device, the combined force decreases sharply when the projectile passes the first orifices and climbs again when the wave front arrives at the rear end of the third cavity. Meanwhile, due to the inflow of some propellant in the barrel gas reflection device, the overall combined force of the artillery equipped with the barrel gas reflection device is smaller than that of the conventional artillery for a period of time after the projectile passes through the orifices, which is also one of the reasons for the low recoil of artillery equipped with the barrel gas reflection device.

**Figure 8:**
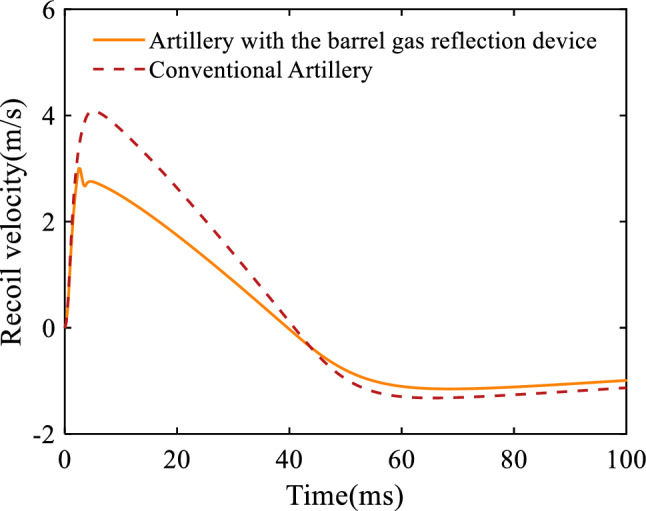
Variation of the recoil velocity with respect to time.

**Figure 9:**
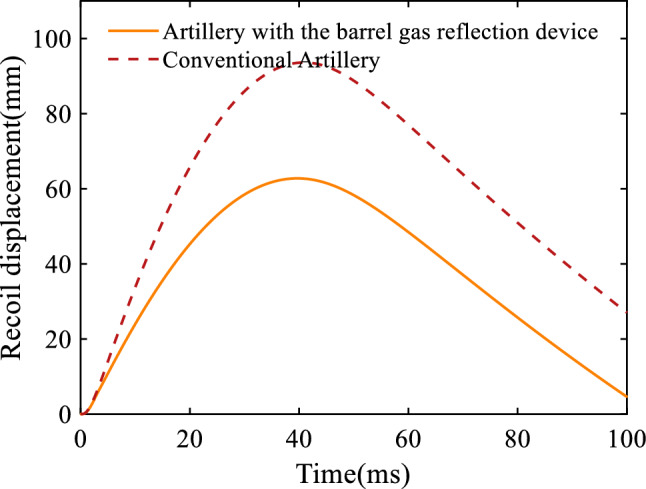
Variation of the recoil displacement with respect to time.

Figure 8 represents the artillery recoil velocity curve, where the positive and negative values of the velocity indicate the direction, and the positive value is the direction of recoil. From the figure, it can be seen that in the initial stage, the velocity of the artillery with the barrel gas reflection device is comparable to that of the conventional artillery. When the projectile crosses the first orifices, the velocity of the artillery with the barrel gas reflection device decreases steeply due to the action of the gas reverse force of the barrel gas reflection device, and the velocity will continue to decrease as the projectile crosses the second and third orifices. Since the after-effect period of the artillery with the barrel gas reflection device is slightly longer than that of the conventional artillery, the artillery decreases more slowly in the later stages of the recoil process. Figure 9 shows the recoil displacement curve of the artillery, from which it can be seen that the maximum recoil displacement of the artillery with the barrel gas reflection device is 62.8 mm, the maximum recoil displacement of the conventional artillery is 93.6 mm, and the recoil reduction efficiency of the barrel gas reflection device can be calculated as 29.62% by using Eq. (25).

The above-analyzing results indicate that the proposed barrel gas reflection device has a high recoil reduction efficiency, while the lower muzzle pressure characteristic of the device implies a lower muzzle hazard, and then the device also has a small effect on the muzzle velocity of the projectile as well as the artillery's rate of fire.

### Parametric sensitivity analysis

Combined with the analysis results of 3.1, it can be seen that the barrel gas reflection device is affected by several design parameters, especially the orifices diameter of the barrel gas reflection device, the location of the orifices, and the length of the barrel gas reflection device. Since the length of each section of the device is constrained by the total length of the device and the location of the second orifices and third orifices is affected by the location of the first orifices as well as the length of the device, to simplify the calculations, all the orifices diameters and the length of each section of the barrel gas reflection device will be considered to be the same, and the diameter of the orifices, the total length of the barrel gas reflection device, and the location of the first orifices will be regarded as the variables. A sensitivity analysis will be conducted on these parameters to investigate the influence of these parameters on the action effect of the barrel gas reflection device.

The Latin hypercube sampling method^22,23^ was used to select 100 sets of parameters within the theoretically permissible limit range of mechanical structures, denoted by ${\mathbf{S}} = \left[ {{\mathbf{s}}_{1} ,{\mathbf{s}}_{2} ,...,{\mathbf{s}}_{100} } \right]$. The parameter ${\mathbf{S}} = \left[ {{\mathbf{s}}_{1} ,{\mathbf{s}}_{2} ,...,{\mathbf{s}}_{100} } \right]$ is brought into the model to get 100 sets of corresponding results, denoted by ${\mathbf{Y}} = \left[ {y_{1} ,y_{2} ,...,y_{100} } \right]$. Table 2 lists the theoretical limit ranges of these parameters.

**Table 2:** The theoretical limit ranges of the parameters.

Item	Upper limit	Lower limit
The orifices diameter of the barrel gas reflection device(mm)	6	14
The location of the first orifices (mm)	200	1400
The length of the barrel gas reflection device (mm)	300	900

According to the Kriging theory^24,25^, the agent model of the action effect of the barrel gas reflection device is established, which is expressed as Eq. (29).

29 $$ y\left( {\mathbf{s}} \right) = \mu \left( {\mathbf{s}} \right) + G\left( {\mathbf{s}} \right) $$

where, $\mu \left( {\mathbf{s}} \right)$ denote the mathematical expectations of $y\left( {\mathbf{s}} \right)$; $G\left( {\mathbf{s}} \right)$ denote a Gaussian process with mathematical expectation 0 and covariance $Cov\left( {G\left( {{\mathbf{s}}_{i} } \right),G\left( {{\mathbf{s}}_{j} } \right)} \right) = \sigma_{G}^{2} R\left( {{\mathbf{s}}_{i} ,{\mathbf{s}}_{j} } \right)$; $\sigma_{G}^{2}$ denote the variance of $G\left( {\mathbf{s}} \right)$;${\mathbf{R}}\left( {{\mathbf{s}}_{i} ,{\mathbf{s}}_{j} } \right)$ denote the Kriging kernel function, which represents the spatial correlation between sample points^26,27^.

The predicted result $\hat{Z}$ of any point ${\mathbf{s}}_{new}$ in the parameter space and predicted variance $s_{{\hat{y}}}^{2}$ of the Kriging model are obtained through optimal linear unbiased estimation by using ${\mathbf{S}} = \left[ {{\mathbf{s}}_{1} ,{\mathbf{s}}_{2} ,...,{\mathbf{s}}_{100} } \right]$ and ${\mathbf{Y}} = \left[ {y_{1} ,y_{2} ,...,y_{100} } \right]$.

30 $$ \hat{Z} = \hat{\mu } + {\mathbf{r}}\left( {{\mathbf{s}}_{new} } \right)^{T} {\mathbf{R}}^{ - 1} \left( {{\mathbf{Y}} - \hat{\mu }} \right) $$

31 $$ s_{{\hat{y}}}^{2} = \hat{\sigma }_{G}^{{^{2} }} \left[ {1 - {\mathbf{r}}\left( {{\mathbf{s}}_{new} } \right)^{T} {\mathbf{R}}^{ - 1} {\mathbf{r}}\left( {{\mathbf{s}}_{new} } \right) + \frac{{\left( {1 - {\mathbf{1}}^{T} {\mathbf{R}}^{ - 1} {\mathbf{r}}\left( {{\mathbf{s}}_{new} } \right)} \right)^{2} }}{{{\mathbf{1}}^{T} {\mathbf{R}}^{ - 1} {\mathbf{r}}\left( {{\mathbf{s}}_{new} } \right)}}} \right] $$

where, $\hat{\mu }$ denote the estimates of pending mathematical expectations;$\hat{\sigma }_{G}^{{^{2} }}$ denote the estimation of variance $\sigma_{G}^{2}$; ${\mathbf{r}}\left( {{\mathbf{s}}_{new} } \right) = \left[ {{\mathbf{R}}\left( {{\mathbf{s}}_{new} ,{\mathbf{s}}_{1} } \right),{\mathbf{R}}\left( {{\mathbf{s}}_{new} ,{\mathbf{s}}_{2} } \right),...,{\mathbf{R}}\left( {{\mathbf{s}}_{new} ,{\mathbf{s}}_{100} } \right)} \right]^{T}$; ${\mathbf{R}}$ denote the correlation coefficient matrix; ${\mathbf{1}}$ denote a 100-row, 1-column matrix with all elements all 1's. $\hat{\mu }$, $\hat{\sigma }_{G}^{{^{2} }}$, ${\mathbf{R}}$ can be obtained by Eqs. (32)–(34).

32 $$ \hat{\mu } = \frac{{{\mathbf{1}}^{T} {\mathbf{R}}^{ - 1} {\mathbf{Y}}}}{{{\mathbf{1}}^{T} {\mathbf{R}}^{ - 1} {\mathbf{1}}}} $$

33 $$ \hat{\sigma }_{G}^{{^{2} }} = \frac{{\left( {{\mathbf{Y}} - {\mathbf{1}}\hat{\mu }} \right)^{T} {\mathbf{R}}^{ - 1} \left( {{\mathbf{Y}} - {\mathbf{1}}\hat{\mu }} \right)}}{100} $$

34 $$ {\mathbf{R}} = \left( {\begin{array}{*{20}c} {{\mathbf{R}}\left( {{\mathbf{s}}_{1} ,{\mathbf{s}}_{1} } \right)} & {{\mathbf{R}}\left( {{\mathbf{s}}_{1} ,{\mathbf{s}}_{2} } \right)} & \cdots & {{\mathbf{R}}\left( {{\mathbf{s}}_{1} ,{\mathbf{s}}_{100} } \right)} \\ {{\mathbf{R}}\left( {{\mathbf{s}}_{2} ,{\mathbf{s}}_{1} } \right)} & {{\mathbf{R}}\left( {{\mathbf{s}}_{1} ,{\mathbf{s}}_{2} } \right)} & \cdots & {{\mathbf{R}}\left( {{\mathbf{s}}_{2} ,{\mathbf{s}}_{100} } \right)} \\ \vdots & \vdots & \ddots & \vdots \\ {{\mathbf{R}}\left( {{\mathbf{s}}_{100} ,{\mathbf{s}}_{1} } \right)} & {{\mathbf{R}}\left( {{\mathbf{s}}_{100} ,{\mathbf{s}}_{2} } \right)} & \cdots & {{\mathbf{R}}\left( {{\mathbf{s}}_{100} ,{\mathbf{s}}_{100} } \right)} \\ \end{array} } \right) $$

**Figure 10:**
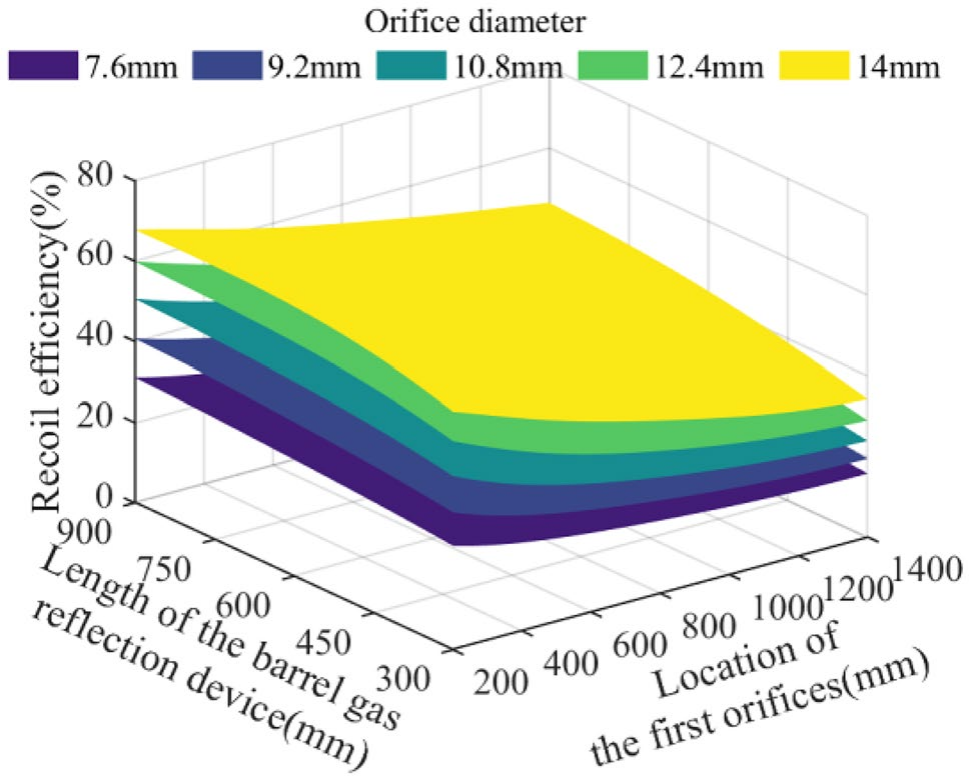
Effect of multi-parameter on the recoil reduction efficiency.

**Figure 11:**
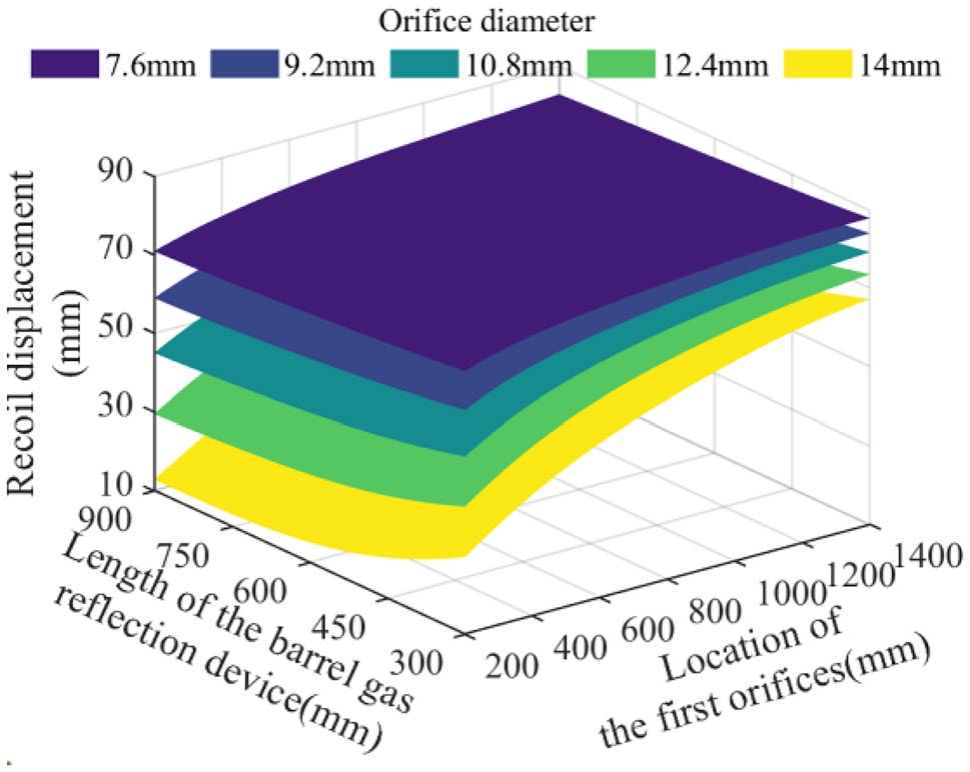
Effect of multi-parameter on the recoil displacement.

**Figure 12:**
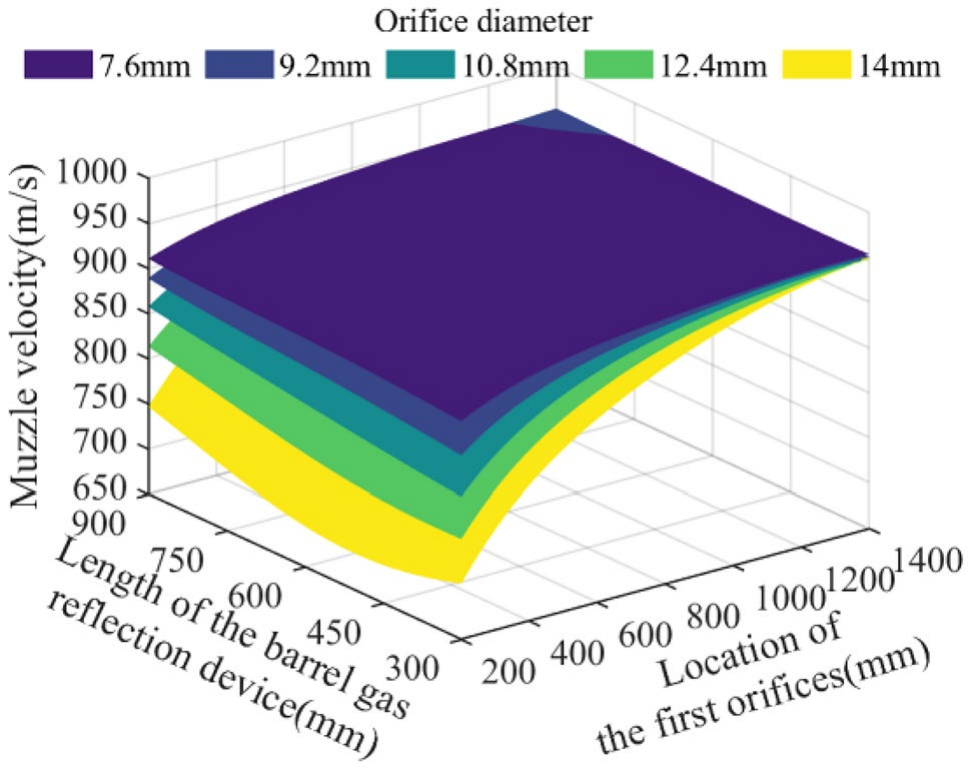
Effect of multi-parameter on the muzzle velocity.

**Figure 13:**
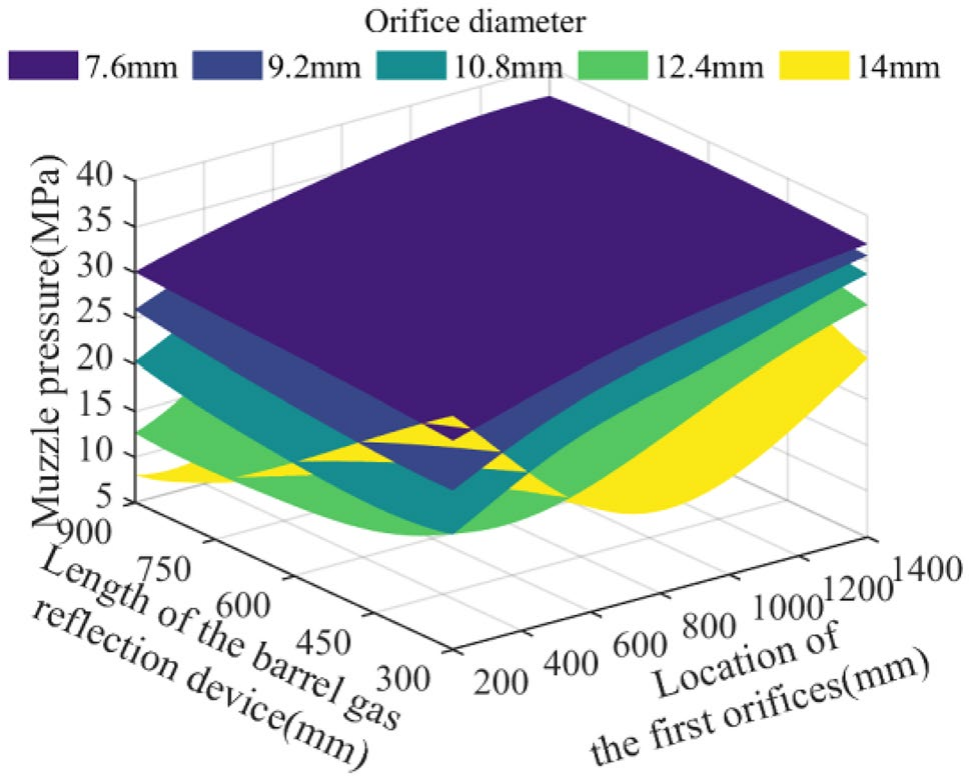
Effect of multi-parameter on the recoil pressure.

In the parameter space of the theoretical limit range, 512,000 sets of parameter combinations are selected and brought into the model of the action effect of the barrel gas reflection device to calculate, and analyze the effects of the key parameters such as the orifice diameter of the barrel gas reflection device, the location of the first orifices, and the length of the barrel gas reflection device on the recoil reduction efficiency of the barrel gas reflection device, the maximum recoil displacement of the artillery, the muzzle velocity of the projectile, and the muzzle pressure of the artillery. The analyzed results are plotted as follows:

The five surfaces in Figs. 10, 11, 12 and 13 represent different orifice diameters, the left horizontal axis represents different lengths of the barrel gas reflection device, the right horizontal axis shows the location of the first orifices, and the vertical axis represents the results of the simulation.

Figure 10 shows that the recoil reduction efficiency of the barrel gas reflection device increases with the increase of the orifice diameter, the growth of the device, and the decrease of the distance between the first orifices and the bottom of the bore. Meanwhile, the recoil reduction efficiency of the device is most sensitive to the change in the orifice diameter, and the maximum theoretical efficiency can reach 67.81%. Figure 11 then represents the effect of each parameter on the recoil displacement of the artillery, through the surface of the figure can be seen that the orifice diameter is small, and the change of each parameter on the recoil displacement of the artillery has a small effect; when the orifice diameter is large, the recoil displacement of the artillery will be reduced with the growth of the device and the decrease of the distance between the first orifices and the bottom of the bore, the artillery's theoretical minimum recoil displacement of 12.93 mm.

Figure 12 represents the effect of each parameter on the projectile muzzle velocity, from which it can be seen that when the location of the first orifices is far away from the bottom of the bore, the projectile muzzle velocity is hardly affected; when the location of the first orifices is close to the bottom of the bore, the projectile muzzle velocity decreases sharply, especially in the case of larger orifice diameter, while the length of the barrel gas reflection device has a smaller effect on the projectile muzzle velocity, and the theoretical projectile muzzle velocity drops to 692.47 m/s at most.

Figure 13 represents the effect of each parameter on the muzzle pressure, it can be seen that in the location of the first orifices far from the bottom of the bore, the device length has less effect on the muzzle pressure, the muzzle pressure almost only with the increase of the orifice diameter and decrease; and with the first orifices location close to the bottom of the bore, the muzzle pressure not only with the increase of the orifice diameter first decreases and then increases, but also with the increase in the length of the device decreases, the theory of the lowest muzzle pressure is 8.04 MPa.

Through the Figs. 10, 11, 12 and 13 comparison analysis, it can be seen that the recoil reduction efficiency of the barrel gas reflection device, the maximum recoil displacement of the artillery, the projectile muzzle velocity, and muzzle pressure is a combination of multiple parameters, the result of mutual influence, there is also coordination and constraints between them, such as when the pursuit of excessive the recoil reduction efficiency of the barrel gas reflection device, the structural parameters obtained tend to be too large an impact on the projectile muzzle velocity at the same time, and the muzzle pressure also did not reach a more optimal result.

## Experiment

**Figure 14:**
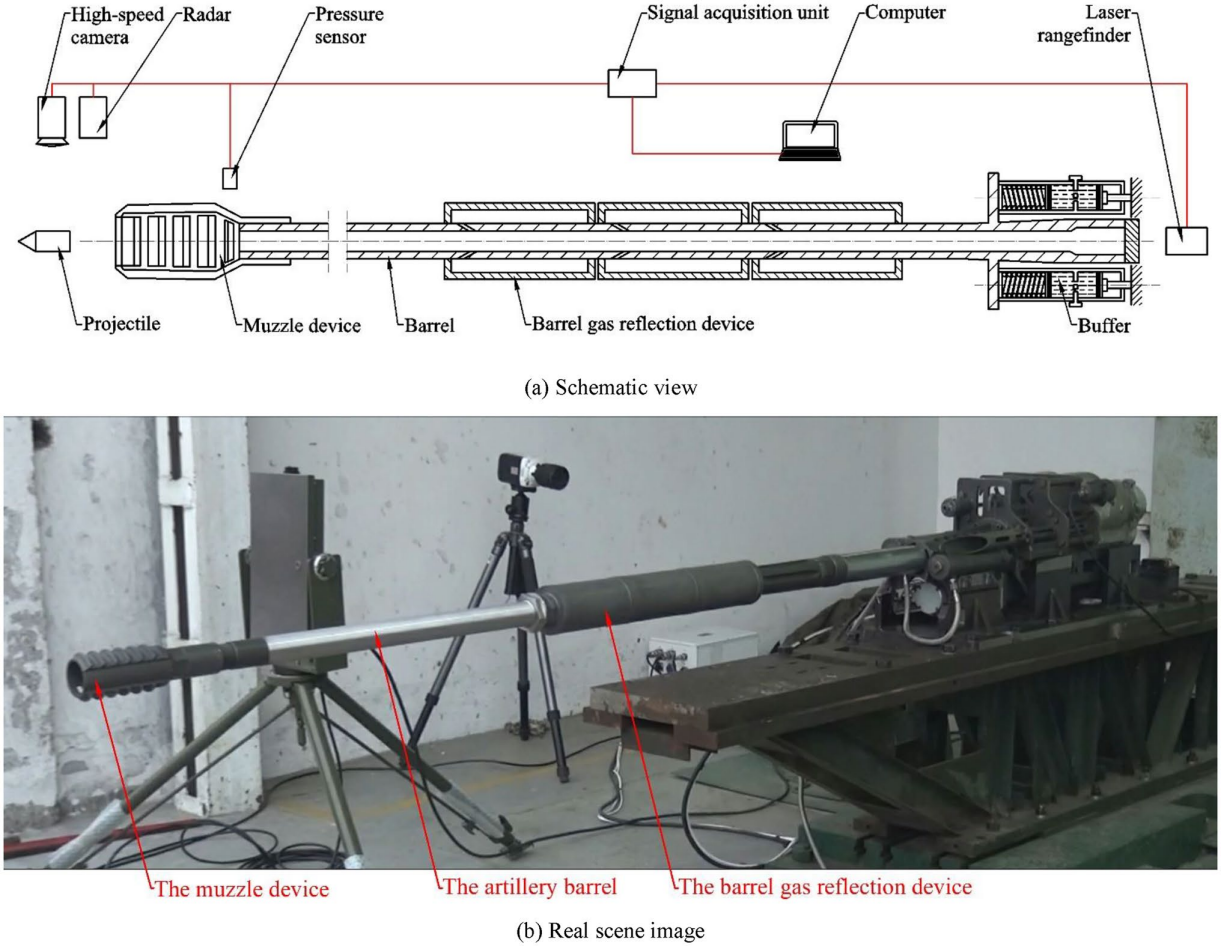
Experiment rig. (**a**) Schematic view. (**b**) Real scene image.

To verify the accuracy of the established model and the effectiveness of the barrel gas reflection device, multiple sets of firing experiments are carried out on a 30-mm caliber artillery platform, and the schematic diagram of the experimental test setup is shown in Fig. 14a. The maximum recoil displacement of the artillery is measured using a laser rangefinder mounted at the end of the artillery, the projectile muzzle velocity is measured using a radar mounted at the front of the muzzle, the muzzle pressure is measured using pressure sensors mounted near the muzzle, and the firing process is filmed using a high-speed camera. All test results are captured by the signal acquisition unit and transmitted to the analysis system. The real scene of the test is shown in Fig. 14b. To achieve the effect of verification and comparison, five groups of tests were conducted as follows:

1. Firing tests of a 30 mm caliber artillery under the condition that neither the muzzle device nor the barrel gas reflection device of the barrel is equipped;
2. Firing tests of a 30 mm caliber artillery under the condition that the muzzle device is not equipped but the barrel gas reflection device is equipped and the orifices diameter of the device is 8 mm;
3. Firing tests of a 30 mm caliber artillery under the condition that the muzzle device is not equipped but the barrel gas reflection device is equipped and the orifices diameter of the device is 10 mm;
4. Firing tests of a 30 mm caliber artillery under the condition that the barrel gas reflection device is not equipped but the muzzle device is equipped;
5. Firing test of a 30 mm caliber artillery under the condition that the muzzle device was equipped together with the barrel gas reflection device, and the orifices diameter of the device are 10 mm.

The rest of the parameters of the 30 mm caliber artillery are shown in Table 1, and for each set of tests, multiple single shots as well as multiple bursts of shots were fired, and the test results were averaged and recorded in Table 3.

**Table 3:** Simulated and experimental results.

Experiment no	The muzzle device	The barrel gas reflection device	Orifice diameter (mm)		Recoil displacement (mm)	Muzzle pressure (MPa)	Muzzle velocity (m/s)	Recoil reduction efficiency (%)
1	Not equipped	Not equipped	–	Simulation	93.6	–	955.06	–
				Experiment	98.5	3.91	959.64	–
				Error	4.97%	–	0.48%	–
2	Not equipped	Equipped	8mm	Simulation	71.2	–	948.15	22.17%
				Experiment	75.2	2.14	953.57	21.90%
				Error	5.32%	–	0.57%	1.23%
3	Not equipped	Equipped	10mm	Simulation	62.8	–	943.62	29.58%
				Experiment	66.3	1.97	947.21	29.34%
				Error	5.28%	–	0.38%	0.82%
4	Equipped	Not equipped	–	Experiment	64.6	4.12	954.42	30.71%
5	Equipped	Equipped	10mm	Experiment	45.8	2.03	931.75	44.54%

By comparing the test results of groups 1, 2, and 3 in Table 3, it can be seen that the maximum recoil displacement is reduced from 98.5 to 75.2 mm and 66.3 mm after the artillery is equipped with the barrel gas reflection device with the orifices diameter of 8 mm and 10 mm, respectively, and the rate of reduction is 23.65% and 32.69%, respectively. The recoil reduction efficiency of the barrel gas reflection device with the orifices diameter of 8 mm and 10 mm are 21.90% and 29.34%, which is a very obvious effect of recoil reduction. In the artillery equipped with the barrel gas reflection device with the orifices diameter of 8 mm and 10 mm after the projectile muzzle velocity of 953.57 m/s and 947.21 m/s, and compared with the artillery not equipped with the barrel gas reflection device the projectile muzzle velocity of 959.64 m/s decreased by 0.63% and 1.30%, respectively. It can be seen that the barrel gas reflection device has little effect on the projectile muzzle velocity. In addition, by comparing the maximum muzzle pressure, after the artillery was equipped with the barrel gas reflection device with the orifices diameter of 8 mm and 10 mm, the muzzle pressure was reduced from 3.91 to 2.14 MPa and 1.97 MPa respectively, with a reduction of 45.27% and 49.46%, which shows that the barrel gas reflection device has a significant effect on reducing the muzzle hazards.

Comparison of the simulation data with the test data in Group 1, 2, and 3 tests in Table 3 also shows that the simulation results are in good agreement with the test results, and the maximum error is only 5.32%, which verifies the accuracy of the proposed model with the barrel gas reflection device.

By comparing the test results of groups 3 and 4 in Table 3, it can be seen that the maximum recoil displacement and recoil reduction efficiency of the artillery equipped with the barrel gas reflection device only and the artillery equipped with the muzzle device only are the same, while the muzzle pressure of the artillery equipped with the barrel gas reflection device only is reduced by 52.18% compared to that of the artillery equipped with the muzzle device only. And by comparing the muzzle flames observed in the tests, the artillery equipped with the barrel gas reflection device only produces almost no gunpowder gas spraying to the side and rear when it fires, which further demonstrates that the barrel gas reflection device is able to satisfy the requirement of high efficiency and low hazard of reducing the recoil force.

By comparing the test results of groups 4 and 5 in Table 3, it can be seen that the barrel gas reflection device and the muzzle device can produce the effect at the same time, and its recoil reduction effect is higher than the effect of a single device, but slightly smaller than the effect of the two devices superimposed on the effect. This is due to the fact that the barrel gas reflection device reduces the muzzle pressure of the artillery, making the recoil reduction effect of the muzzle device less effective.

## Conclusions

This paper proposes a high-efficiency and low-hazard recoil reduction technology and a principle model of the barrel gas reflection device is established based on the technology. Through the theoretical analysis and experimental analysis of the principle model of the barrel gas reflection device, the following conclusions are summarized (Supplementary Information S1):

1. Using the coupled internal ballistic model, the flow equations of the barrel gas reflection device, and the kinetic equations of the artillery, a theoretical model of the firing process of the artillery with the barrel gas reflection device is established, and the accuracy of the model is verified by comparing it with the experimental results, with the maximum error of 5.32%, which lays a foundation for the design and research of the barrel gas reflection device;
2. Through the theoretical model, the theoretical recoil reduction efficiency of the barrel gas reflection device can reach a maximum of 67.81%, and the muzzle pressure can be reduced by a maximum of 79.51%; at the same time, through the actual firing test, the artillery equipped with the barrel gas reflection device has the same recoil reduction effect as the artillery equipped with the muzzle device, with a significantly lower muzzle hazard, which demonstrates that the high-efficiency and low-hazard recoil reduction technology based on the barrel gas reflection effectively solves the contradiction between high-efficiency recoil reduction and low muzzle hazards;
3. The barrel gas reflection device can produce effects with the muzzle device at the same time, and it has little effect on the velocity of the projectile muzzle velocity, which shows that the high-efficiency and low-hazard recoil reduction technology based on barrel gas reflection can be applied to most of the artilleries, and it provides a new way of thinking for the development of the future artillery recoil reduction technology.

### Supplementary Information

Supplementary Information.

## Data Availability

The datasets used and/or analysed during the current study available from the corresponding author on reasonable request.

## List of symbols

$C_{p}$: The specific heat at constant pressure
$C_{V}$: The specific heat at constant volume
$D$: The outer diameter of the barrel gas reflection device
$D_{i}$: The outer diameter of the artillery barrel
$e_{0}$: Thickness of the propellant
$e_{i}$: The energy per unit mass of the propellant gas through the orifices of the ith cavity
$e_{k}$: The energy per unit mass of the propellant gas through the orifices of the muzzle
$f$: The propellant force
$F_{0}$: The initial force of the spring
$F_{hz}$: The recoil force of the artillery equipped with the barrel gas reflection device
$F_{hz0}$: The recoil force of the conventional artillery
$G\left( {\mathbf{s}} \right)$: A Gaussian process with mathematical expectation 0 and covariance $Cov\left( {G\left( {{\mathbf{s}}_{i} } \right),G\left( {{\mathbf{s}}_{j} } \right)} \right) = \sigma_{G}^{2} R\left( {{\mathbf{s}}_{i} ,{\mathbf{s}}_{j} } \right)$
$k$: The specific heat ratio
$k_{0}$: The stiffness of the spring
$l_{0}$: The equivalent length of the chamber
$$l_{g}$$: Barrel length
$l_{i}$: The distance between the orifices of the ith cavity and the chamber throat
$l_{\psi }$: The equivalent length of the free chamber volume
$le_{i}$: The length of the ith cavity of the barrel gas reflection device
$m$: The projectile mass
$m_{0}$: The mass of the propellant gas in the artillery bore
$m_{i}$: The mass of the propellant gas in the ith cavity of the barrel gas reflection device
$n$: Burning rate-pressure exponent
$n_{1}$: The number of cavities
$p_{0}$: Average bore pressure
$p_{i}$: The average pressure in the ith cavity of the barrel gas reflection device
$q_{i}$: The flow rate per second through the orifices of the ith cavity
$q_{k}$: The flow rate per second through the orifices of the the muzzle
$R$: The gas constant
${\mathbf{R}}$: The correlation coefficient matrix
${\mathbf{R}}\left( {{\mathbf{s}}_{i} ,{\mathbf{s}}_{j} } \right)$: The Kriging kernel function
${\mathbf{S}}$: The simulation parameter set
${\mathbf{s}}_{i}$: The ith set of simulation parameters
${\mathbf{s}}_{new}$: A point in the parameter space
$s_{{\hat{y}}}^{2}$: The predicted variance of the Kriging model
$S_{0}$: The cross-section area of the artillery bore
$S_{i}$: The equivalent area of the ith orifices
$$t$$: Time
$T_{0}$: The temperature of the propellant gas in the artillery bore
$T_{i}$: The temperature of the propellant gas in the ith cavity of the barrel gas reflection device
$u_{0}$: Burning rate coefficient
$U_{i}$: The potential energy of the propellant gas in the ith cavity of the barrel gas reflection device
$v_{0}$: Velocity of the projectile
$v_{00}$: The muzzle velocity of the conventional artillery
$v_{k}$: Muzzle gas velocity
$V_{0}$: The chamber volume
$V_{i}$: The volume of the propellant gas in the ith cavity of the barrel gas reflection device
$x_{0}$: The travel of the projectile respectively
$x_{i}$: The displacement of the wave front in the ith cavity of the barrel gas reflection device
$X$: The recoil displacement of the artillery equipped with the barrel gas reflection device
$X_{0}$: The recoil displacement of the conventional artillery
${\mathbf{Y}}$: The simulation result set
$y_{i}$: The simulation result of the ith set
$y\left( {\mathbf{s}} \right)$: The predicted results of parameters ${\mathbf{s}}$
$z$: The relative burned thickness of the propellant
$z_{k}$: The max relative burned thickness of the propellant
$\hat{Z}$: The predicted result

## Greek symbol

$\alpha$: Covolume of the propellant gas
$\beta$: Coefficient in after effect period
$\sigma_{G}^{2}$: The variance of $G\left( {\mathbf{s}} \right)$
$\hat{\sigma }_{G}^{{^{2} }}$: The estimation of variance $\sigma_{G}^{2}$
$\chi$: Form characteristic parameters of the propellant
$\chi_{s}$: Form characteristic parameters of the propellant
$$\Delta$$: Loading density
$\varphi$: Coefficient of the secondary works in the artillery bore
$\eta$: Recoil reduction efficiency
$\lambda$: Form characteristic parameters of the propellant
$\mu$: Form characteristic parameters of the propellant
$\mu_{0}$: Discharge coefficient of the orifices
$\mu_{k}$: Discharge coefficient of the muzzle
$\mu_{s}$: Form characteristic parameters of the propellant
$\mu \left( {\mathbf{s}} \right)$: The mathematical expectations of $y\left( {\mathbf{s}} \right)$
$\hat{\mu }$: The estimates of pending mathematical expectations
$\theta$: A parameter about the specific heat ratio
$\rho_{0}$: The density of the propellant gas in the artillery bore
$\rho_{i}$: The density of the propellant gas in the ith cavity of the barrel gas reflection device
$\rho_{p}$: Density of the propellant
$$\omega$$: Charge mass
$\psi$: Relative burned mass of the propellant

## References

[1] Bhatnagar RM (2005). Recoil motion theorem. Proc. Inst. Mech. Eng. Part K J. Multi-Body Dyn..

[2] Wu YC, Chang H, Tsung TT (2011). Dynamic characteristics of a recoil system when firing projectiles with Mach 4.4 muzzle velocity from a 105 mm cannon. J. Test. Eval..

[3] Harinder JS, Norman MW (2014). Optimal control of gun recoil in direct fire using magnetorheological absorbers. Smart Mater. Struct..

[4] Ouyang Q, Zheng J, Li Z, Hu M, Wang J (2016). Controllability analysis and testing of a novel magnetorheological absorber for field gun recoil mitigation. Smart Mater. Struct..

[5] Luo Y, Xu D, Li H (2020). Analysis of the dynamic characteristics of the muzzle flow field and investigation of the influence of projectile nose shape. Appl. Sci..

[6] Zhao W, Hou BL, Bao D (2023). Multi-parameter identification of soft recoil artillery launch process using IICCA. J. Vib. Shock.

[7] Kathe, E. Rarefaction wave gun propulsion. Troy, New York, USA: Rensselaer Polytechnic Institute (2002).

[8] Qin ZL, Li XX, Guan HT, Liang W (2022). Research on the stability control strategy of the aerial gun servo system with continuous firing. J. Ordnance Equip. Eng..

[9] Zhang X, Wang Y (2010). Analysis of dynamic characteristics for rarefaction wave gun during the launching. J. Appl. Mech. Trans. ASME.

[10] Cho HC, Yoon JK, Shin HD (1999). Unsteady flow analysis of combustion processes in a Davis gun. J. Inst. Energy.

[11] Nuri, Y. O., & Sam, L. Recoilless rifle weapon systems. U. S. Dept. of Defense, Army Materiel Command (1976).

[12] Zhang, F., Liao, Z., Liu, G., & Chen, Y. Numerical investigation on reversely jet low recoil gun propulsion. In *International Conference on Mechanical Engineering and Mechanics*, pp. 320–323 (2007).

[13] Cao, Y., Zhang, X., Li, Z., & Wu, X. Modeling and Simulation of back blast flow field of a recoilless gun launching in finite space. In *International Conference on Material Science and Civil Engineering*, pp. 526–531 (2016).

[14] Wiri S, Ritter AC, Bailie JM (2017). Computational modeling of blast exposure associated with recoilless weapons combat training. Shock Waves.

[15] Eckehard B (2006). Dynamical loading of the muzzle area of a gun barrel including a muzzle brake. J. Pressure Vessel Technol. Trans. ASME.

[16] Cayzac R, Carette E, Roquefort TAD, Renard FX, Roux D, Balbo P, Patry JN (2011). Computational fluid dynamics and experimental validations of the direct coupling between interior, intermediate and exterior ballistics using the euler equations. J. Appl. Mech. Trans. ASME.

[17] Zhang H, Chen Z, Jiang X, Li H (2013). Investigations on the exterior flow field and the efficiency of the muzzle brake. J. Mech. Sci. Technol..

[18] Wang DY, Nan FQ, Liao X, Xiao ZL, Du P, Wang BB (2022). Analysis of dynamic characteristics for rarefaction wave gun during the launching. Acta Armamentarii.

[19] Li PF, Zhang XB (2020). Numerical research on the impinging effect of sequential muzzle blast waves formed by successive shooting at high frequency. Propell. Explos. Pyrotech..

[20] Jin ZM (2004). Interior ballistics of guns.

[21] Tao C, Zhang Y, Li S, Jia C, Li Y, Zhang X, He Z (2010). Mechanism of interior ballistic peak phenomenon of guns and its effects. J. Appl. Mech. Trans. ASME.

[22] Helton JC, Davis FJ (2003). Latin hypercube sampling and the propagation of uncertainty in analyses of complex systems. Reliab. Eng. Syst. Saf..

[23] Olsson A, Sandberg G, Dahlblom O (2003). On Latin hypercube sampling for structural reliability analysis. Struct. Saf..

[24] Irfan K (2005). Application of kriging method to structural reliability problems. Struct. Saf..

[25] Kaymaz I, Mcmahon CA (2005). A response surface method based on weighted regression for structural reliability analysis. Prob. Eng. Mech..

[26] Zhan D, Qian J, Cheng Y (2016). Pseudo expected improvement criterion for parallel EGO algorithm. J. Glob. Optim..

[27] Peng X, Lin C, Yizhong MA (2022). Kriging adaptive modeling and global optimization algorithm based on weighted expectation infill criterion. Comput. Integr. Manuf. Syst..

